# Traumatic neuroma of the mandible: A case report with spontaneous remission

**DOI:** 10.4317/jced.51659

**Published:** 2014-07-01

**Authors:** Bruno C. Jham, Nádia L. Costa, Aline C. Batista, Elismauro F. Mendonça

**Affiliations:** 1 DDS, MS, PhD. College of Dental Medicine – Illinois, Midwestern University; 2 DDS, MS, PhD. Department of Oral Pathology, School of Dentistry, Federal University of Goiás; 3 DDS, MS, PhD. Head and Neck Department, Araújo Jorge Hospital

## Abstract

Traumatic neuroma is a well-known disorder involving peripheral nerves, which occurs following trauma or surgery. The lesion develops most commonly in the soft tissues of the mental foramen area, lower lip and tongue. Intra-osseous lesions arising in jawbones are very uncommon. In this paper, we report a new case of an intra-osseous traumatic neuroma, discovered incidentally on a panoramic radiograph obtained for orthodontic documentation. In addition, the case herein described developed spontaneous remission, a situation not previously reported in the literature. Finally, we discuss relevant demographic, clinical, microscopic, immunohistochemical and treatment aspects of traumatic neuromas.

** Key words:**Amputation neuroma, traumatic neuroma, mandible, spontaneous remission.

## Introduction

Traumatic neuroma is a well-known disorder involving peripheral nerves, which occurs following trauma or surgery (1). The lesion is not a true neoplasm; rather, it represents a frustrated attempt at nerve reparation and an exaggerated response to injury, consisting of reactive hyperplasia of the nerve tissue (1-3).

Traumatic neuromas may develop in any region of the body, including the head and neck (2). In this region, they have been reported to develop more frequently following parotidectomy and neck dissection (1). Intraorally, the lesion develops most commonly in the mental foramen, lower lip and tongue. Intra-osseous lesions arising in jawbones are very uncommon and it has been suggested that neuromas may not readily develop in bone due to pressure from the surrounding tissues (4). The treatment of traumatic neuromas is surgical excision, with no recurrence expected (3).

The aim of this paper is to report a new case of an intra-osseous traumatic neuroma, discovered incidentally on a panoramic radiograph obtained for orthodontic documentation. In addition, the case herein described developed spontaneous remission, a situation not previously reported in the literature.

## Case Report

**Figure 1:**
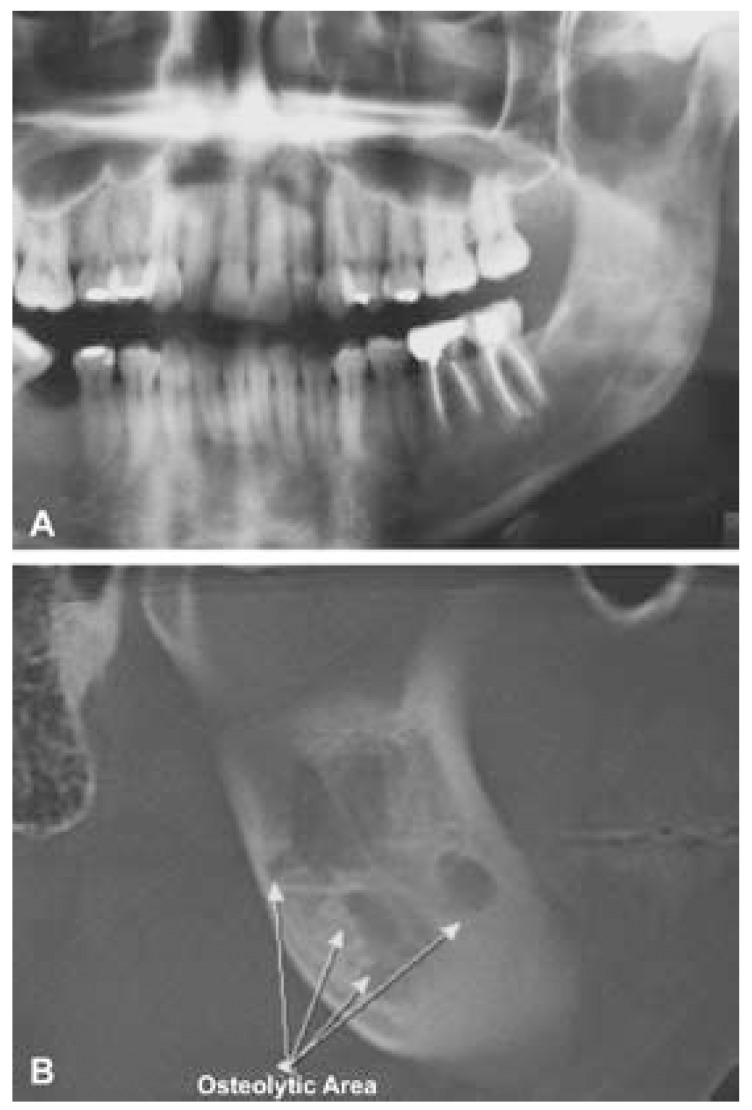
A. Panoramic radiographic images showing an osteolytic ill-defined lesion on the left side of the mandible, extending from the second premolar to the mandibular ramus, and; B. Cone beam CT scan in section images showing osteolytic lesion and destruction of the mandibular vestibular bony cortical.

**Figure 2:**
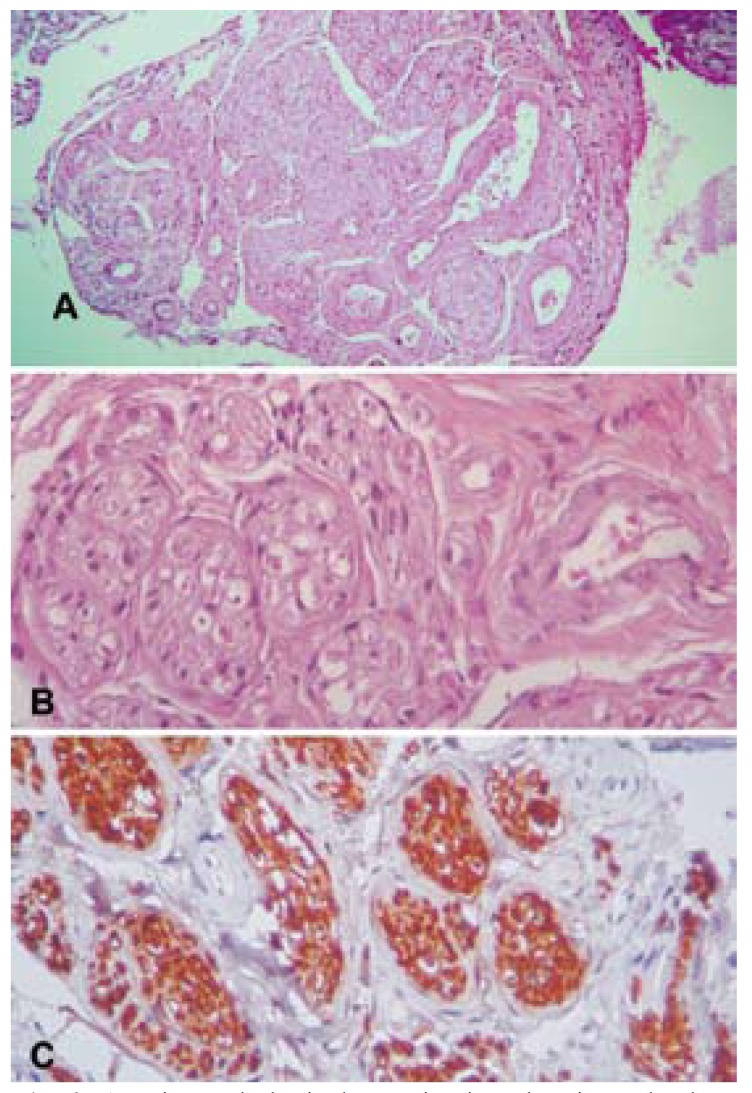
A. Histopathological examination showing a haphazard, tortuous proliferation of nerve bundles within a vascularized fibrous connective tissue stroma. Hematoxylin and eosin stain, original magnification x10; B. Cross-sectioned nerve bundle within a vascularized fibrous connective tissue stroma. Hematoxylin and eosin stain, original magnification x40; C. Strong positivity of the bundles for the neural marker S-100. Immunohistochemical staining, original magnification x40.

**Figure 3:**
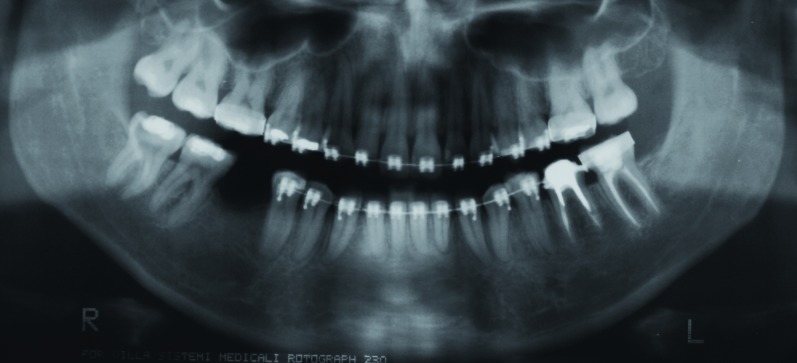
Panoramic radiograph showing total regression, one year after the incisional biopsy.

A 22-year-old white female was referred to the Oral Medicine Service of the Araujo Jorge Hospital for evaluation of an osteolytic, mandibular lesion detected on a panoramic radiograph obtained for orthodontic documentation purposes. During anamnesis the patient reported a previous history of complicated exodontias of an unerupted left mandibular third molar two years previously. Medical and familial histories were unremarkable. Extraoral and intraoral examinations were within normal limits. Radiographic examination revealed an ill-defined, multilocular lesion on the left side of the mandible, extending from the second premolar to the mandibular ramus (Fig. 1). No expansion of the cortical bone was observed. A computed tomography confirmed the panoramic radiograph findings, showing a non-expansible lytic lesion. Based on clinical and radiographic observations, the initial diagnosis was traumatic bone cyst. The patient was scheduled for surgical removal of the lesion, but failed to attend and was lost to follow-up. Five years later, the patient reappeared seeking care for the same lesion and seeking orthodontic and implants treatment. New panoramic radiograph and cone beam computed tomography were requested and the images showed a lesion with the same characteristics cited previously (Fig. 1). An incisional biopsy was performed and histopathological examination revealed a haphazard, tortuous proliferation of nerve bundles within a vascularized fibrous connective tissue stroma (Fig. 2). Immunohistochemical analysis was carried out and showed a strong positivity of the bundles for the neural marker S-100 (Fig. 2). The tissue was also stained for the proliferation marker Ki-67; only a few positive cells were observed, underlying the lesion’s low proliferative index. The final diagnosis was traumatic neuroma. We chose to follow the patient closely and, surprisingly, after three months a new panoramic radiograph revealed initial regression of the lesion. After one year, total regression was observed (Fig. 3). The patient was followed for one additional year, with no lesion recurrence being observed, before being lost to follow-up.

## Discussion

Traumatic neuromas are uncommon lesions. In 2005, Jones and Franklin reviewed 44,007 oral and maxillofacial specimens and found 149 cases [0.34%] (5). Salla *et al*. [2009] found only 15 cases among 21,476 specimens [0.07%] diagnosed in an oral pathology service (6). Importantly, only soft tissue traumatic neuromas were seen in the latter study, demonstrating that intraosseous lesions, such as the one herein presented, are even less common than their soft tissue counterpart. Indeed, in a study with almost 50,000 oral cavity biopsy specimens, only 11 cases [0.02%] of intrabony traumatic neuromas were found (7).

Traumatic neuromas can occur at any age, but are most often diagnosed in young and middle-aged adults (5,7). Women tend to be more affected than men, with an estimated female-to-male ratio of 2:1 (5). In agreement with the literature, our patient was a young adult female. Clinically, soft tissue traumatic neuromas present as smooth-surfaced, non-ulcerated nodules, most commonly in the mental foramen, lower lip and tongue (3). Intraosseous traumatic neuromas present as unilocular or multilocular radiolucent defects. The most common location is the posterior mandible, due to damage of the alveolar inferior nerve following tooth extraction or sagittal ramus split during osteotomy (3). Similarly, a previous third molar extraction could have been the triggering factor in the current case. Lesions arising in the maxilla are extremely rare, with only two cases reported in the literature (8,9). Thus, the differential diagnosis of intraosseous traumatic neuromas includes a wide variety of lesions that have a predilection for the posterior mandible, such as odontogenic and non-odontogenic tumors and cysts (3). The most common symptom of traumatic neuromas is pain, due to compression of nerves by the tumor, although studies indicate it is present in only 25-30% of the cases (4,7). Further, pain seems to be more common in females (8). In agreement with the literature, our patient presented with a painful, multilocular radiolucency in the posterior mandible. Other symptoms of intra-oral traumatic neuromas include anesthesia and paresthesia (2,3). Importantly, a malignancy should be ruled out if a patient presents with paresthesia, since it may be the first symptom of metastasis in 30% of patients (10).

Histopathologically, traumatic neuromas typically present as non-encapsulated lesions, containing a large amount of haphazardly arranged nerve fascicles, within a densely collagenous and fibroblastic stroma. Occasionally, scar tissue may also be seen (11). Immunohistochemical studies may be needed to achieve the final diagnosis, with S-100 being the single best antibody, whereas antibodies to EMA, CD57, and collagen IV are of secondary value (12). In our study, we used S-100 to confirm the neural origin of the lesion and Ki-67 to confirm its low proliferative index and benign nature.

The treatment of choice is nerve sparing surgical excision. An optimal technique, with minimal manipulation and severance of nerve fiber, is essential for adequate outcome. Other second-line therapeutic options found in the literature are stereotactic radiosurgery, local infiltration of steroids, sympathetic ganglion block, percussion, and ultrasonic therapy (2,3). In the case herein reported, it was opted to keep the patient under close follow-up and this ultimately revealed the most striking aspect of our case – spontaneous remission. To our knowledge, this is the first report of spontaneous regression of an intraosseous traumatic neuroma. However, this phenomenon has been observed in other neural tumors; it is estimated that between 4-16% of vestibular schwannomas [a true benign neoplasm, also known as acoustic neuroma] will regress spontaneously (13,14). Proposed mechanisms for spontaneous involution of vestibular schwannomas – which could also possibly explain the case we report – include ischaemic necrosis secondary to intratumoral thrombosis and immunologically mediated apoptosis (14,15).
